# Exploiting the STAT3 Nexus in Cancer-Associated Fibroblasts to Improve Cancer Therapy

**DOI:** 10.3389/fimmu.2021.767939

**Published:** 2021-11-11

**Authors:** Amr Allam, Marina Yakou, Lokman Pang, Matthias Ernst, Jennifer Huynh

**Affiliations:** Olivia Newton-John Cancer Research Institute and La Trobe University School of Cancer Medicine, Heidelberg, VIC, Australia

**Keywords:** STAT (signal transducer and activator of transcription), tumor development, cancer associated fibroblasts (CAF), cytokines, tumor microenvironment

## Abstract

The tumor microenvironment (TME) is composed of a heterogenous population of cells that exist alongside the extracellular matrix and soluble components. These components can shape an environment that is conducive to tumor growth and metastatic spread. It is well-established that stromal cancer-associated fibroblasts (CAFs) in the TME play a pivotal role in creating and maintaining a growth-permissive environment for tumor cells. A growing body of work has uncovered that tumor cells recruit and educate CAFs to remodel the TME, however, the mechanisms by which this occurs remain incompletely understood. Recent studies suggest that the signal transducer and activator of transcription 3 (STAT3) is a key transcription factor that regulates the function of CAFs, and their crosstalk with tumor and immune cells within the TME. CAF-intrinsic STAT3 activity within the TME correlates with tumor progression, immune suppression and eventually the establishment of metastases. In this review, we will focus on the roles of STAT3 in regulating CAF function and their crosstalk with other cells constituting the TME and discuss the utility of targeting STAT3 within the TME for therapeutic benefit.

## The STAT3 Signaling Pathway

**Figure 1:**
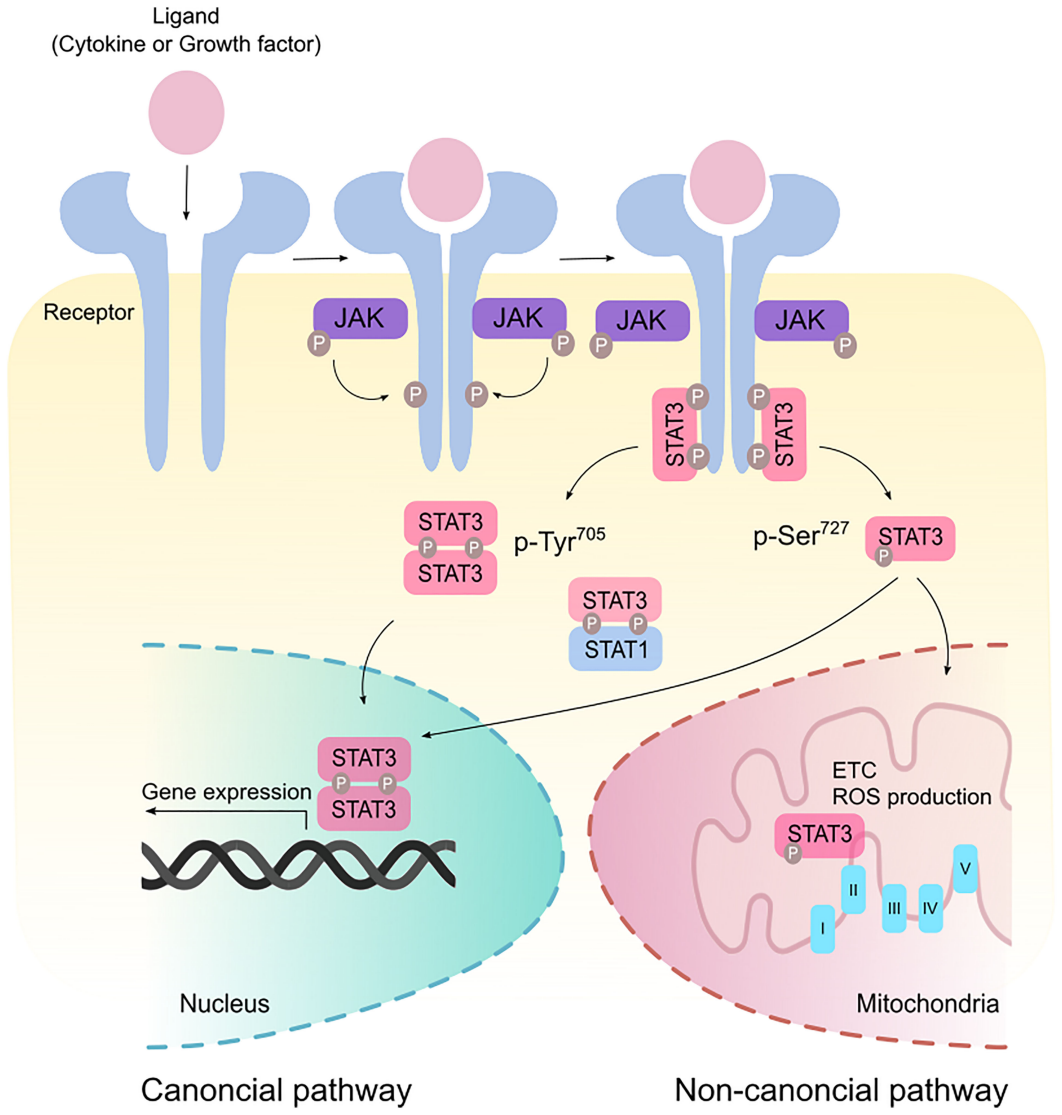
Canonical and non-canonical STAT3 signaling. Binding of ligands such as cytokines and growth factors to their cognate receptors stimulate receptor dimerization and recruitment of Janus kinase (JAK). JAK phosphorylates the cytoplasmic tails of the receptor to create a docking site for STAT3. In the canonical pathway, STAT3 is phosphorylated at the Tyr^705^ residue and form homodimers or STAT3:STAT1 heterodimers that modulate gene expression in the nucleus. Maximal transcriptional activation can also be induced by the non-canonical activation of STAT3 *via* phosphorylation at the Ser^727^ residue. p-Ser^727^ STAT3 can then translocate to the mitochondria to regulate the electron transport chain (ETC) and production of reactive oxygen species (ROS).

Signal transducer and activator of transcription 3 (STAT3) was originally coined as acute-phase response factor (APRF) when it was first identified as a DNA-binding protein downstream of the interleukin (IL)-6 cytokine (1, 2). STAT3 shares structural similarities with the other 6 members of the STAT proteins containing an amino terminus, a coiled-coil domain, a DNA-binding domain, a SH2-domain, and a transcription activation domain. Among the major cytokines that act upstream of STAT3 are members of the IL-6 family. Canonical STAT3 signaling involves glycoprotein 130 (GP130) receptor homodimerizing with ligand-bound receptor leading to the recruitment of Janus kinases (JAKs) to facilitate STAT3 phosphorylation (**Figure 1**). This signaling cascade is negatively regulated by suppressor of cytokine signaling 3 (SOCS3) which binds simultaneously to JAK and Y757 on GP130 (3). Such binding non-competitively inhibits JAK activity independently of ATP. Indeed, mice containing a knock-in phenylalanine mutation at Y759 (equivalent to Y757 in humans) disrupting SOCS3-binding results in the spontaneous development of gastric adenomas in a cytokine-dependent manner, highlighting the oncogenic potential of dysregulated STAT3 activity (4). Adding to another layer of negative regulation, GP130 is ubiquitinated by the c-Cbl E3 ligase in a ligand-dependent manner resulting in its lysosomal degradation (5). Upon STAT3 Y705 phosphorylation, STAT3 forms homodimers or STAT3:STAT1 heterodimers, enabling its active nuclear translocation and binding to a palindromic DNA consensus sequence. While Y705 phosphorylation necessitates the transcriptional activities of STAT3, further S727 phosphorylation potentiates maximal transcriptional activation (6). In addition to its prototypical roles in transcription, STAT3 can regulate metabolism following S727 phosphorylation where it translocates to the mitochondria and modulates electron transport and reactive oxygen species production (7).

STAT3 signaling and its transcriptional outputs are integral to normal biological processes and maintenance of homeostasis as *Stat3*-deficient mice are embryonic lethal (8). STAT3 co-ordinates many of the tightly regulated processes that underpin the wound healing response to restore epithelial integrity following barrier disruption and dysfunction (9). Yet deviation of these processes is frequently observed in solid malignancies and is in part attributed to aberrant STAT3 activity in stromal cancer-associated fibroblasts. Fibroblasts are critical mediators of all stages of the wound healing response by virtue of their ability to produce, remodel and contract extracellular matrix (ECM), in addition to the production of growth factors and their pro-angiogenic properties (10). However, when unchecked STAT3 can exploit the wound healing characteristics of cancer-associated fibroblasts (CAFs) to sculpt a tumor milieu that is conducive to fibrosis, cancer cell migration and dissemination, while limiting immune cell-infiltration and responsiveness to therapy.

## CAFs in Tumor Development

During the wound healing and regenerative response, normal fibroblasts play a critical role in maintaining tissue homeostasis after injury, where they trans-differentiate into a subtype of fibroblasts called myofibroblasts which induce force-mediated contractility and the deposition of ECM components, such as collagen I–IV, XVIII, proteoglycans, glycosaminoglycans (GAGs) and hyaluronic acid (HA) (10, 11). Myofibroblasts can break down fibrin clots and remodel collagens to eventually promote wound closure. In addition, activated fibroblasts in a wound healing setting recruit immune cells to the site of injury to fight any infections and promote proliferation (12).

For a long time, cancer progression was thought to be primarily driven by cells that acquire oncogenic mutations leading to their transformation into malignant cells. However, it is now appreciated that non-malignant cells within the tumor microenvironment (TME) play equally important roles in driving the development and progression of tumors (13, 14). Owing to the complexity and heterogenous nature of tumors, different approaches have been employed to target various facets of the TME in an effort to modulate the extracellular matrix (ECM), cytokines that drive chronic inflammation, hypoxia and angiogenesis (reviewed in (14–20)). In addition, other strategies home into targeting the cellular components of the TME including stromal and immune cells. Blocking CAF activity and subsequently the recruitment and differentiation of tumor-promoting immune cells such as macrophages and myeloid-derived suppressor cells (MDSCs) correlate with better patient prognosis in many solid malignancies including pancreatic, colorectal, gastric, ovarian, prostate, and squamous cell carcinoma cancers [reviewed in (21–24)]. These seminal findings highlight the importance of targeting different compartments of the TME in combination with conventional therapies for best possible patient treatment outcome.

In the context of cancer, activated fibroblasts transform into CAFs where their functions are exploited by tumor cells within the TME. It is unclear if the transformation of normal fibroblasts into CAFs is due to the acquisition of genetic mutations. However, it is well-established that inflammatory cytokines (e.g. IL-1, IL-4, IL-5, IL6, IL-8, IL-10, IL-11 and IL-17) (12), vitamin A and D deficiency (25, 26), stromal stiffness and mechanical forces exerted on normal fibroblasts in the TME, are all vital factors in driving this transformation (27, 28). CAFs are a key cellular component in the TME and play an essential role in promoting favorable conditions for tumor cell survival and proliferation (23). CAFs remodel the TME through excessive production and transforming of ECM components, production of cytokines and growth factors, which together impact normal resident and tumor cells. CAFs are typically categorized into two major classes based on their functions. Fibroblasts which induce high levels of ECM remodeling and participate in fibrotic tissue formation are termed myofibroblastic CAFs (myoCAFs) (29). Immunomodulatory fibroblasts are called inflammatory CAFs (also known as iCAFs) and produce an array of inflammatory cytokines including those from the IL-6 family of cytokines (e.g., IL-6 and IL-11), which are key upstream effectors for STAT3 signaling (30). MyoCAFs are characterized by high expression of alpha smooth muscle actin (αSMA), fibroblasts activation protein (FAP) and low IL-6 expression, while iCAFs typically harbor low levels of αSMA and high IL-6 expression (30). Although myoCAFs and iCAFs are the most studied subtypes of CAFs, new emerging subtypes have been reported, including antigen presenting CAFs (apCAFs) and vascular CAFs (vCAFs) (23, 31–33). apCAFs are characterized by their surface expression of major histocompatibility complex II (MHC-II), which has been shown to have immuno-suppressive effects. vCAFs reside in the microvascular regions and are characterized by the expression of melanoma adhesion molecule (MCAM; also called CD146) and IL-6. vCAFs promote human intrahepatic cholangiocarcinoma through IL-6/IL-6R crosstalk with tumor cells (34).

Recently, a wealth of evidence underscores the ability for CAFs to modulate immune responses within the TME [reviewed in (35, 36)]. Despite the general consensus that CAFs confer pro-tumorigenic effects, emerging literature alludes to an anti-tumor role for CAFs albeit the molecular mechanisms underpinning this process remain unclear (37, 38). In this review we will focus on the pro-tumorigenic effects of CAFs and how the transcription factor STAT3 modulates the tumor-promoting activities of CAFs in the TME. Emerging evidence points towards a role for STAT3 in modulating CAF activities in the TME (39–44). Here, we will focus on how STAT3 signaling regulates CAF function, and to what extent does this play a role in ECM remodeling and mediating intercellular crosstalk within the TME to create favorable conditions for tumor progression and subsequent metastasis.

## Effects of STAT3 Signaling on CAFs and ECM Remodeling

In normal tissue, remodeling of the ECM is in large dependent on resident fibroblasts which maintain the structural integrity of the ECM *via* the secretion of ECM components, including collagens, tenascin, periostin and proteases (23). Collectively, these components provide the ECM with its unique biochemical and biomechanical properties, which subsequently modulate the behavior of other tissue resident cells (11, 45).

**Figure 2:**
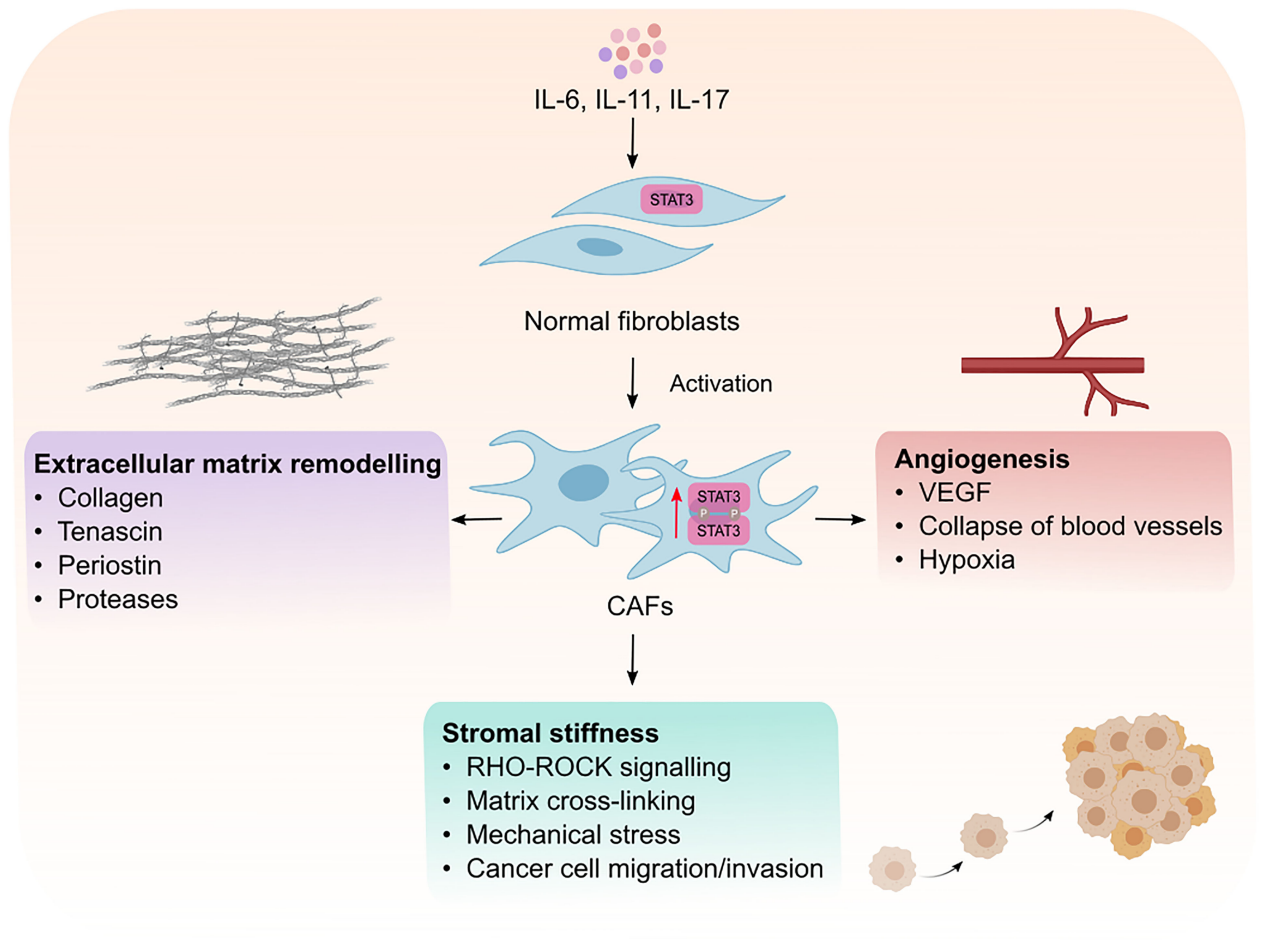
Intrinsic effects of STAT3 signaling in CAFs. Cytokines such as IL-6, IL-11 and IL-17 mediate the phosphorylation and activaton of STAT3 resulting in the transformation of normal fibroblasts into cancer associated fibroblasts (CAFs). Activated CAFs can then remodel the extracellular matrix, promote matrix cross-linking leading to stromal stiffness and mechanical stress. Mechanical stresses in the stroma consequently leads to the collapse of blood vessels and induction of hypoxia which creates an environment permissible to tumor development. CAFs can also promote tumor vascularization which facilitates the migration and invasion of cancer cells to distant sites. VEGF; vascular endothelial growth factor.

Intrinsic STAT3 activity in CAFs has emerged as a mechanism by which CAFs support tumor progression (**Figure 2**). It has been shown that activation of STAT3 in CAFs promotes the production of pro-tumorigenic factors including IL-6, VEGF and TGF-β, suggesting that STAT3 activation is a key feature of activated CAFs (46, 47). Supporting this hypothesis, high STAT3 activity in CAFs correlates with poor patient prognosis in colorectal cancer and inactivation of STAT3 reduces tumor burden in a murine model of inflammation-associated colon cancer (43). It remains to be determined if STAT3 activation is a shared feature in activated CAFs across all cancers. Activated CAFs undergo epigenetic modifications which trigger uncontrolled actomyosin contractility leading to stromal stiffness. Long-term exposure to leukemia inhibitory factor (LIF, member of IL-6 cytokine family) induces STAT3 acetylation which leads to an epigenetic loss of the Src homology region 2 domain-containing phosphatase 1 (SHP-1) (48). SHP-1 is a tumor suppressor, and its loss of expression is frequently observed in many cancers including hepatocellular carcinoma, leukemia, and lymphoma (49–51). Importantly, SHP-1 is a negative regulator of JAK1/STAT3 signaling, and its loss induces constitutive activation of JAK1/STAT3 *via* GP130, in turn, up-regulating actomyosin contractility *via* phosphorylation of the regulatory myosin light-chain 2 (MLC2) through the RHO-ROCK pathway (48, 52). Actomyosin promotes force-mediated matrix remodeling, which is characterized by excessive deposition of ECM components such as collagen and fibronectin, which promotes stromal stiffness and fibrosis (53, 54).

Interestingly, force-mediated matrix remodeling induces mechanical stress which in turn potentiates a positive feedback loop for CAF activation, leading to an increase in stromal stiffness, irreversible matrix cross-linking and excessive fibrotic reaction, also known as desmoplasia. Among other solid malignancies, desmoplasia is a hallmark of pancreatic cancer and plays an integral role in blocking immune cell infiltration and mediating chemoresistance (55). Although, there is no evidence for a direct link between STAT3 activation and fibrosis in cancer models, it has been shown that pharmacological inhibition of STAT3 reduces and lowers the incidence of fibrotic tissue formation in a mouse model of colitis (56). Moreover, Papaioannou and colleagues showed that STAT3 binds to the enhancer of the collagen type 1α2 subunit, which encodes the *COL1A2* gene. COLA12 is essential for collagen deposition by human lung myofibroblasts (40), which highlights the role of STAT3 in collagen deposition. Indeed, the same authors showed that pharmacological inhibition STAT3 lowered the ability of myofibroblasts to produce collagen I and remodel the ECM. Furthermore, IL-11, an upstream effector of STAT3 and member of the IL-6 family of cytokines, was reported to induce fibrosis in different fibrotic diseases including, idiopathic pulmonary fibrosis and systematic sclerosis (57, 58). Therefore, it would not be surprising if STAT3 plays a role in promoting fibrosis in cancer, however, this warrants further investigation.

CAFs produce matrix proteases which remodel the ECM, forming tracks within the TME to allow tumor cell migration and invasion. In addition, CAFs also promote angiogenesis and neovascularization to allow tumor cell dissemination from the primary tumor site (59). Although CAFs can promote angiogenesis *via* expressing vascular endothelial growth factor (VEGF), CAFs can also induce angiogenesis by secreting IL-11, subsequently leading to STAT3 activation in human umbilical vein endothelial cells in a VEGF-independent manner (60). Furthermore, CAFs can promote epithelial-to-mesenchymal transition in lung cancer cells through induction of matrix proteases (MMP-2, MMP-9) and VEGF in response to IL-6/STAT3 signaling, which subsequently leads to ECM remodeling and angiogenesis (41). IL-6 neutralizing antibodies inhibit the expression of MMP-2, MMP-9 and VEGF which indicates the importance of IL-6/STAT3 signaling for their expression. Thus, strong evidence suggests STAT3 activity is a key modulator of CAF function and their ability to produce and remodel the ECM which helps sculpt an environment permissible to tumor growth and spread.

## STAT3 Mediates Crosstalk Between CAFs and Tumor Cells

**Figure 3:**
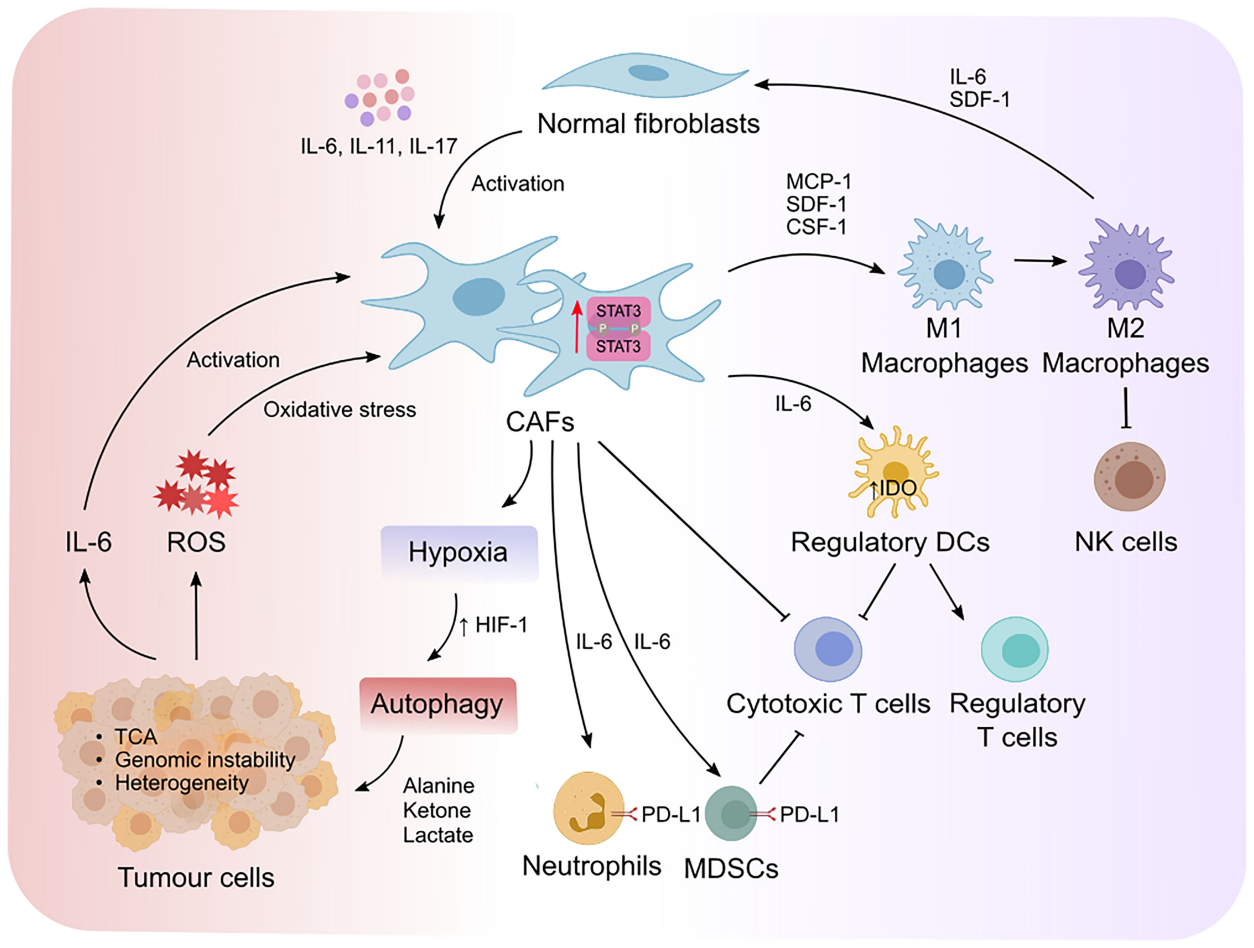
STAT3 mediates reciprocal crosstalk between CAFs, tumor cells and immune cells in the TME. Cytokine-mediated activation of STAT3 promotes the transformation of normal fibroblasts to cancer associated fibroblasts (CAFs). STAT3 increases the expression of hypoxia-inducible factor-1 (HIF-1) that leads to hypoxia and autophagy. This results in the production of high energy products that fuel the tricarboxylic (TCA) cycle in tumor cells, as well as the production of IL-6 that form a positive feedback loop to enable further activation of CAFs. Moreover, elevated oxidative stress induced by tumor-derived reactive oxygen species (ROS) can induce genomic instability and promote tumor heterogeneity. On the other hand, STAT3-mediated activation of CAFs exert immunosuppressive effects *via* the recruitment and polarization of macrophages from an M1 to an M2 endotype, which suppresses the cytotoxic activity of natural killer (NK) cells. Moreover, CAFs recruit regulatory dendritic cells (DCregs) to inhibit the activation of cytotoxic T cells while simultaneously promoting the proliferation of regulatory T cells. IL-6/STAT3 signaling in CAFs also promotes the development of myeloid-derived suppressor cells (MDSCs) and induces PD-L1 expression in neutrophils. Collectively, deviation of cytokine mediated STAT3 activity in the TME alters the metabolic landscape of tumors and fosters an immunosuppressive environment that can evade immune clearance.

Crosstalk between CAFs and tumor cells is essential for tumor progression which is in part dictated by intrinsic STAT3 activity in CAFs (**Figure 3**). CAFs can promote the proliferation and survival of tumor cells *via* the release of growth factors, cytokines, and exosomes (12, 61). Moreover, activated CAFs can promote the formation of fibrotic tissue, which acts as a physical barrier against chemotherapy and immune cell infiltration. Fibrotic tissue is also stiff in nature and can lead to the collapse of blood vessels creating a low glucose and nutrient environment for tumor cells, which is a prominent feature in pancreatic cancers (55, 62). Therefore, the metabolite exchange between tumor cells and CAFs is essential for tumor cell survival and proliferation (63, 64). CAFs that undergo autophagy can supply nutrients required by tumor cells. Interestingly, IL-6 and IL-17 which act upstream of STAT3 can promote autophagy in CAFs (65–67). Moreover, STAT3 was shown to induce the expression of hypoxia-inducible factor (HIF)-1, a transcription factor which is induced during hypoxia (68), in esophageal squamous cell carcinoma (ESCC) *via* binding to its promoter (69). These observations implicate a potential contribution of the STAT3 signaling axis in hypoxia-induced autophagy in CAFs however this is yet to be investigated. CAF-induced autophagy results in the production of high energy metabolites, including alanine, ketone and lactate, which fuel the tricarboxylic cycle in tumor cells (64, 70, 71). In response to this, tumor cells produce more IL-6 and IL-8 thereby fueling a feed-forward loop and enabling a continuous supply of nutrients from adjacent CAFs (72–74). Inhibition of IL-6 or IL-8 using neutralizing antibodies significantly reduced CAF-induced autophagy in mouse xenograft models of head and neck squamous cell carcinoma (72), supporting a role for IL-6 and IL-8 in autophagy. CAF-induced autophagy is also triggered by HIF-1-induced oxidative stress. Tumor cell-driven reactive oxygen species (ROS) released by tumor cells induced oxidative stress in CAFs (72). Interestingly, elevated oxidative stress in mouse embryonic fibroblasts promotes STAT3 phosphorylation and its translocation to the nucleus independently of cytokines, which may subsequently promote tumor cell survival. Moreover, the ROS/STAT3 signaling axis has been reported to induce tumor progression in pancreatic, prostate and liver cancers (39, 75, 76). These findings indicate that STAT3-dependent CAF-induced autophagy in response to oxidative stress is imposed by tumor cells. Reciprocally, CAFs with high oxidative stress can induce high levels of genomic instability in tumor cells *via* a bystander effect, promoting tumor heterogeneity and a more aggressive phenotype (73).

CAFs can produce an array of growth factors and cytokines (TGF-β, FGFs, HGH, IL-6 and LIF) providing strong evidence for paracrine signaling between CAFs and tumor cells which is integral to tumorigenesis (36, 61). Interestingly, the spatial distribution of CAFs in terms of their localization, relative to tumor cells within the TME, confers nuanced differences in their phenotypic transformation. For instance, CAFs adjacent to tumor cells retain a myCAF phenotype, while iCAFs tend to reside distal from tumor cells within the TME in pancreatic cancer (77). The crosstalk between CAFs and tumor cells in the TME plays a critical role in regulating CAFs phenotypic changes within the TME. Indeed, tumor cells produce high levels of TGF-β to induce αSMA expression in adjacent fibroblasts and their transformation into myofibroblasts. In addition, tumor cell-derived TGF-β suppresses IL-6 expression in adjacent fibroblasts which in turn, inhibits NF-κB signaling (77). These observations suggest that the crosstalk between tumor cells and adjacent fibroblasts dictates the phenotype of CAFs which is also tightly regulated by their spatial location within the TME. IL-6/STAT3 signaling promotes an immunosuppressive CAF phenotype (discussed in detail in the following section), known as iCAFs. iCAFs largely reside at the periphery of tumors (77),, suggesting that the IL-6/STAT3 signaling axis in iCAFs not only plays a role in promoting immunosuppression but also limiting immune cells infiltration towards the tumor core and allowing tumor cells to evade the immune system.

Fibroblast growth factors (FGFs) released by CAFs are another key mechanism by which STAT3 modulates the crosstalk between CAFs and tumor cells. It has been shown that induction of STAT3 *via* FGFR2/STAT3 signaling axis correlated with more aggressive breast cancer (78). In addition, activation of STAT3 *via* FGFR induced accumulation of hyaluronan, an ECM component involved in regulating cellular proliferation and migration as well as the onset of metastasis (79). Moreover, the same study found that inhibition of STAT3, compromised the growth of FGFR-driven tumors and decreased levels of hyaluronan in the TME.

## STAT3 Mediates the Crosstalk Between CAFs and Immune Cells

The ability of cancer cells to evade detection and clearance by the immune system is critical for their survival and progression, as highlighted by the success of immunotherapy. Neoplastic cells utilize various subversive mechanisms to avoid immune-mediated anti-tumor responses such as the suppression of antigen presentation (i.e., MHC expression), “exhausting” immune cells *via* immune checkpoints and the recruitment of immunosuppressive cells (T regulatory lymphocytes) (80, 81). Tumors also engage CAFs in the TME to support immune evasion, allowing tumor cells to disseminate from the primary site and metastasize to distant sites. The immunosuppressive effects of CAFs broadly impact cells of the innate and adaptive immune system, including dendritic cells (DC), macrophages, neutrophils, mast cells, natural killer cells (NK) and T lymphocytes (82–86) which have been reviewed in (36, 87–90).

CAFs can recruit monocytes, macrophages and mast cells *via* the release of monocyte chemotactic protein-1 and stromal cell-derived factor-1 (SDF-1) (also known as CXCL12) (91) (**Figure 3**). Activated macrophages exist on a spectrum of phenotypes ranging from classically activated M1- to alternatively activated M2 macrophages. M2 macrophages display anti-inflammatory, immune-suppressive and tumor-permissive endotypes, while M1 macrophages confer pro-inflammatory, immune-permissive anti-tumor responses (22). STAT3 has been shown to promote M2 macrophage polarization in part due to its intrinsic activity in CAFs. Likewise, SDF-1 and IL-6 released by CAFs and tumor cells in prostate cancer, promotes the polarization of macrophages into an M2 phenotype (92). CAFs also recruit macrophages to the tumor niche *via* macrophage colony stimulating factor (CSF-1) which induces M2 macrophage polarization (93, 94). CAF-induced M2 macrophage polarization *via* the release of STAT3 upstream effectors (e.g., IL-6, CSF-1 and SDF-1) underpins the role that STAT3 activation plays in regulating CAF-macrophage crosstalk and the subsequent immunosuppressive effects of M2 macrophages on other immune cells. Consistent with this, CAF-induced M2 macrophage polarization suppressed NK cell-mediated immune responses in colorectal cancer (95). Reciprocally, M2 macrophages promote the transformation of normal fibroblasts into CAFs *via* IL-6 and SDF-1 in prostate cancer (96). Interestingly, high estrogen-alpha (ER-α) expressing CAFs inhibited tumor progression in prostate cancer, and lowered IL-6 expression in CAFs and macrophages in co-culture, suggesting that IL-6 promotes M2 polarization and the pro-tumor effects of CAFs (97).

DCs present antigen to T cells *via* the expression of MHC-I and -II which in turn triggers an effective immune response (98). Although the crosstalk between CAFs and DCs remains largely unclear, emerging studies show that CAFs support regulatory functions of DCs rather than immune costimulatory functions in hepatocellular carcinoma (86). Regulatory DCs (DCregs) are characterized by high expression of inhibitory molecules such as PD-L1, which suppress effector T cell activation and proliferation (98). In addition, DCregs produce indoleamine-2, 3-dioxygenase (IDO) and other metabolites to induce the proliferation of T regulatory cells (Tregs), which is a T cell subpopulation that dampens cytotoxic T cell responses (99). IDO is an immunosuppressive enzyme which regulates degradation of the essential amino acid tryptophan and triggers cellular stress in response to pro-inflammatory stimulation [reviewed in (100, 101)]. CAF-mediated IL-6 production has been shown to the up-regulate IDO expression in DCs (86). IL-6 neutralizing antibodies and STAT3 inhibitors blocked the ability for CAFs to modulate the function of DCregs. These findings indicate that CAF-DC crosstalk *via* IL-6/STAT3 promotes immunosuppression and tumor progression *via* either directly suppressing effector T cell activation or indirectly by promoting Treg expansion and subsequent effector T cell inactivation.

CAFs also exploit immune checkpoint proteins to suppress anti-tumor cytotoxic T cells and NK cells. For instance, CAFs support the development of MDSCs in pancreatic, colorectal, and liver cancer through induction of STAT3 in response to IL-6 (102–105). Activation of STAT3 in MDSCs and M2 macrophages promotes PD-L1 expression, which in turn inhibits T cell effector function (105–107). In addition, CAFs induce PD-L1 expression in neutrophils in hepatocellular carcinoma in response to IL-6/STAT3 signaling (84). CAFs also express PD-L2 and FASL which suppresses T cell anti-tumor responses (108). Although STAT3 is a key modulator for PD-L2 and FASL in tumor cells, it remains unclear if STAT3 is the key transcription factor that modulates PD-L2 and FASL expression in CAFs (109, 110). Altogether, these findings highlight how STAT3 signaling can impart an immunomodulatory effect in CAFs during tumor development.

## Major Challenges Associated With Studying STAT3 Biology in CAFs

Most experimental studies employ ambiguous cell surface markers including platelet-derived growth factor receptor (PDGFRα and PDGFRβ), αSMA, vimentin and fibroblast activation protein (FAP) to enrich for CAFs by flow cytometry (111). Other CAF biomarkers have been reported including soluble factors (IL-6, IL-11, TGF-β), ECM components and extracellular vesicles as previously reviewed in (112). In addition, some studies also use negative selection against epithelial (EpCAM), endothelial (CD31), and immune cell markers (CD45). Despite this, these methods also capture normal fibroblasts as they share many surface markers with their cancer-associated counterparts. For instance, while SMA and IL-6 expression can distinguish between myCAFs and iCAFs as discussed previously, these markers may also not resolve other heterogeneous and transcriptionally distinct CAF subpopulations (31) such as apCAFs or vCAFs which express high levels of IL-6 and activate STAT3 in tumor cells as observed in cholangiocarcinoma (34). It is anticipated that single cell sequencing and digital spatial profiling will aid the characterization of CAF subpopulations as well as identification of robust and specific markers to circumvent the current challenges we face. Overall, advances in CAF classification and identification will be key to elucidating the full extent of CAFs in cancer biology and what CAF phenotypes are modulated by STAT3 activity.

Compounding the lack of specific markers, assessing CAF functions *in vitro* also presents with limitations. Firstly, viable CAFs are notoriously difficult to isolate from tumors in sufficient numbers for *in vitro* analysis even for stroma-rich cancers like pancreatic cancer. However, studies have reported successful enrichment of CAFs *via* negative selection of cells positive for epithelial, endothelial and immune cell markers (34). Secondly, isolated fibroblasts and CAFs can change and lose their phenotype when cultured *in vitro* and are particularly sensitive to prolonged passaging in culture. These observations are not entirely surprising, because physiological conditions are hard to faithfully replicate in culture, and CAFs adapt to the dynamic changes of the TME. One method of overcoming this challenge is to culture fibroblasts in 3-dimensional to mimic a more physiologically relevant environment akin to their “natural environment” that affects CAF proliferation, attachment, migration, and elongation (113, 114). Moreover, cancer and stromal cells can be co-cultured in 3-dimensional matrices to capture the crosstalk that occurs in the TME (55, 115, 116). This is relevant to studying STAT3 biology given its pertinent roles in CAF function and CAF-tumor cell crosstalk as previously discussed. Collectively, these *in vitro*-based models bypass the caveats associated with 2-dimensional cultures and attempt to re-create tumor-stroma crosstalk as observed under physiological conditions.

Due to the lack of CAF-specific gene drivers, it is also difficult to lineage trace or conditionally knock out genes in transgenic mouse models. Nevertheless, various inducible transgenic mice have been generated to delete *Stat3* expression in fibroblast and CAF populations which at least provide us some insight into how STAT3 regulates these cell types *in vivo*. For instance, transgenic mice with the Cre recombinase expression under the control of the *Col1a2* promoter to selectively delete *Stat3* have been reported (117) and indicate that CAFs promote colitis-associated colorectal cancer in a STAT3-dependent manner (43). *Pdgfra*-cre mice also exist (23) to selectively delete genes in CAFs however directed ablation of STAT3 in these mice have not been reported to date. Meanwhile, Schaefer and colleagues have recently characterized a role for IL-11 in fibrosis using *Col1a2*-*Cre*ERT, *Il11ra1^l^* ^oxP/^ *^lox^* ^P^ mice as well as directed *Il11* transgene expression in *Col1a2*-*Cre*ER *: Rosa26-Il11* mice (118, 119). Intriguingly, IL-11 drives fibrosis in the lung, heart, and kidney *via* non-canonical ERK but not STAT3 signaling contrary to Chakraborty’s findings (117) which instead suggest canonical STAT3 signaling drives fibroblast function and fibrosis. Taken together, it is important that appropriate *in vitro* assays and murine models are employed to study CAFs in tumor development as well as understanding the influence STAT3 has on CAF functionality. In addition, the discovery of emerging CAF-specific driver genes will facilitate the generation of novel transgenic mice and enable validation studies to ascertain the biological effects STAT3 exerts on the CAF population.

The categorical definition of CAFs is a major area of contention due to their phenotypic heterogeneity, lack of fibroblast-specific cell surface markers, and the limitations of assays employed to functionally characterize CAFs. Understanding and experimentally identifying the full spectrum of CAF phenotypes will be key in mapping out how STAT3 activity contributes to each of their unique subtypes and plastic states. While it is clear that STAT3 activity supports tumor promoting “fibroblast-like” activity, it remains to be reconciled whether the cells assayed in studies thus far are purely reflective of CAFs. As discussed, many studies rely only on one method of identification, typically using non-specific markers which could also enrich for other mesenchymal cell types and cancer cells that have undergone epithelial-to-mesenchymal transition. Moreover, emerging roles for CAFs in anti-tumor responses have been observed highlighting their complex and dichotomous nature. This underscores the importance of establishing a standardized and robust method of studying CAFs to properly inform how we can exploit STAT3-targeting therapies that target specific CAF subpopulations to tip the balance towards an effective anti-tumor response.

## Targeting CAFs in Cancer and the Implications for STAT3-Targeting Therapies

As outlined previously, the mutual relationship between CAFs, tumor cells and immune cells fuels cancer development, immune evasion, and resistance to therapy. This bi-directional crosstalk is facilitated by the secretion of various soluble factors such as cytokines, chemokines, and growth factors which together with CAF cell surface markers, present an opportunity to develop and test their therapeutic intervention in combination with immunotherapies as summarized in **Table 1**.

**Table 1:** Clinical trials targeting CAFs and STAT3 in cancer.

Target	Cancer	Drug Name	Combination Therapy	Current Status	Clinical Trials Identifier
***Depleting CAFs***					
**FAP**	Breast cancer Head & neck cancer	RO6874281	Trastuzumab/Cetuximab	Phase I; active, not recruiting	NCT02627274
	Advanced or metastatic melanoma	RO6874281	Pembrolizumab	Phase I; active, not recruiting	NCT03875079
***Blocking CAF activation***					
**FGFR**	Advanced urothelial cancer	Futibatinib	Pembrolizumab	Phase II; recruiting	NCT04601857
**TGF-β**	Rectal adenocarcinoma	Galunisertib	Chemotherapy & radiotherapy	Phase II; recruiting	NCT02688712
**CXCL12**	Advanced pancreatic cancer	Plerixafor	Cemiplimab	Phase II; recruiting	NCT04177810
***Blocking ECM production and remodelling***					
**MMP9**	Glioblastoma	GS-5745	Bevacizumab	Phase I; not yet recruiting	NCT03631836
**Collagen I production**	Advanced pancreatic cancer	Losartan	Nivolumab & Chemotherapy	Phase II; recruiting	NCT03563248
***Reprogramming CAFs into normal fibroblasts***					
**Vitamin D receptor**	Advanced pancreatic cancer	Paricalcitol	Gemcitabine	Phase II; recruiting	NCT03520790
***STAT3 Inhibitors***					
**STAT3 mRNA**	Advanced, solid tumors & non-small cell carcinoma	Danvatirsen	Durvalumab &/or chemotherapy	Phase IB/II; active, not recruiting	NCT03421353
	pancreatic cancer & mismatch repair deficient colorectal cancer	Danvatirsen	Durvalumab	Phase II; active, not recruiting	NCT02983578
**IL-6 activity**	Late-stage melanoma	Tociluzumab	Ipilimumab and Nivolumab	Phase I; recruiting	NCT03999749
	Prostate cancer	Tociluzumab	Atezolizumab	Phase II; recruiting	NCT03821246
	Metastatic HER2-positive breast cancer	Tociluzumab	Trastuzumab and chemotherapy	Phase I; completed	NCT03135171

One strategy to target CAFs is through the blockade of growth factors that lead to their activation. As mentioned above, TGF-β produced by tumor cells can activate CAFs and promote tumor development. A phase II clinical trial is currently exploring the use of Galunisertib, a TGF-β inhibitor, in combination with a chemotherapy and radiotherapy regime to treat rectal adenocarcinoma (NCT02688712) (**Table 1**). In addition, the FGFR receptor inhibitor, Futibatinib, is being tested in a Phase II clinical trial for its activity in combination with the anti-PD-1 antibody, Pembrolizumab, for the treatment of advanced urothelial carcinoma (NCT04601857).

Another approach to target CAFs is by intercepting their ability to produce and remodel the ECM (120, 121) which would in effect, dismantle the physical barrier that prevents immune cells from penetrating tumors and compromise the scaffold that would otherwise support tumor cell-CAF crosstalk. Blocking the action of MMPs poses another attractive strategy to target CAF-induced remodeling of the ECM. Despite their anti-cancer potential, over 50 MMPs have been tested and failed in clinical trials (122). Failure of MMP inhibitors to confer objective responses in patients is largely due to their lack of specificity, where most MMP inhibitors tested are broad-spectrum. However, ongoing clinical trials are still testing their efficacy particularly in combination with other therapies. For instance, the anti-MMP-9 monoclonal antibody, GS-5745, is in Phase I testing in combination with immunotherapy (Bevacizumab) for the treatment of glioblastoma (NCT03631836).

The marked heterogeneity of the CAF population is another modality that can be targeted to specifically limit pathogenic subsets of CAFs as is the case for cells that highly express FAP (123, 124). FAP-targeting therapies have entered phase I clinical testing including RO6874281, an anti-FAP interleukin-2 variant (125). RO6874281 is currently being assessed as both a single agent and in combination with Trastuzumab in breast cancer patients or Cetuximab in head and neck cancer patients (NCT02627274). Another clinical trial is testing the utility of combining RO6874281 with the immune checkpoint inhibitor, Pembrolizumab, for the treatment of advanced melanoma (NCT03875079).

Blocking soluble factors produced from CAFs could also achieve clinical benefit in patients. For instance, CAFs produce CXCL12 which allow cancer cells to evade detection and clearance by T cells in preclinical studies (126, 127). A phase II clinical trial is testing the efficacy of Plerixafor, a CXCR4 receptor antagonist which blocks the action of CXCL12, in combination with an anti-PD-1 antibody, Cemiplimab, for the treatment of patients with metastatic pancreatic cancer (NCT04177810).

Fibroblasts are programmed to become CAFs within the TME, and hence, there are promising approaches targeting these phenotypic changes by reprograming CAFs (128). An example of these targets include, a Phase II clinical trial currently testing Losartan, an angiotensin inhibitor, in combination with immunotherapy (Nivolumab), and chemotherapy for the treatment of advanced pancreatic cancer (NCT03563248) (121). Angiotensin inhibitors block signals which promote fibroblast activity such as angiotensin II (AngII) through AngII receptor type 1 which in turn, inactivate and reduce the number of CAFs (128). Consistent with these observations, a link has been ascribed for Ang II and IL-6, whereby Ang II can induce IL-6 expression and contributes to vascular disease and hypertension (129). Thus, by targeting STAT3 signaling or its upstream cytokine IL-6, Ang II may also be blocked and in turn, reprogram CAFs into a quiescent form. Furthermore, vitamin D deficiency has also been associated with increased fibrosis and aggressive tumorigenesis. The supplementation of vitamin D subsequently inhibits tumor development and enhances the delivery of chemotherapies into the tumor in pre-clinical models (130, 131). In line with this, a phase II clinical trial is currently underway testing paricalcitol, a vitamin D analogue, in combination with a standard chemotherapy program of Gemcitabine and nab-paclitaxel for metastatic pancreatic adenocarcinoma patients (NCT03520790).

A number of clinical trials are assessing the safety and efficacy on STAT3 inhibitors (**Table 1**). Considering the pivotal role STAT3 plays in promoting CAF transformation and their pro-tumorigenic functions, therapeutically targeting STAT3 signaling provides an opportunity to indirectly target CAF function. Moreover, the prospect of combining STAT3 inhibitors with other therapies has utility as evident by active clinical trials assessing the safety and efficacy of STAT3 inhibitors in combination with immunotherapies in various cancers (**Table 1**). One such example is Danvatirsen, an antisense oligonucleotide used to target STAT3 mRNA. The utility of combining Danvatirsen with Durvalumab, a PD-L1 inhibitor, alone or in combination with chemotherapy is currently being tested in a clinical trial for the treatment of advanced, solid tumors and non-small cell carcinoma (NCT03421353). Another phase II clinical trial is also testing Danvatirsen in combination with Durvalumab but for pancreatic cancer and mismatch repair deficient colorectal cancer (NCT02983578). As previously described, blocking IL-6 activity would be another way to target STAT3 and CAFs function and this could be achieved with Tocilizumab, a monoclonal antibody against the IL-6 receptor (132). Phase I/II clinical trials are currently recruiting patients to test the benefit of combining Tociluzumab with various immunotherapies for the treatment of late-stage melanoma (NCT03999749), prostate cancer (NCT03821246), and metastatic HER2-positive breast cancer (NCT03135171). It remains to be seen whether these combinatorial approaches provide patient benefits, and whether objective responses are certainly due to the direct modulation of STAT3-dependent CAF function, or a more likely combination of effects on other cells in the TME. Nevertheless, the number of clinical trials is indicative of the potential for STAT3-targeting therapies to target pathogenic CAFs and improve the efficacy of immunotherapies.

## Conclusion

It is apparent STAT3 is a key molecular driver of CAF function and dictates the crosstalk between cells of the TME to foster tumor development and metastatic spread. The reciprocal relationship between CAFs, tumor cells and immune cells is made possible through the release of soluble factors including the IL-6 family of cytokines which further reinforces a feed forward loop. Evidently, targeting the activities of STAT3 and its associated cytokines have shown promising results in patients and improved the efficacy of immune checkpoint inhibition. Yet it remains unclear how much CAFs contribute to these objective responses in patients. Moreover, the implementation of STAT3-targeting therapies must consider the marked heterogeneity of CAFs and they should be tailored towards suppressing tumor-promoting populations while preserving those that do not contribute to disease to essentially promote an anti-tumor response in the TME. Nevertheless, blocking STAT3 activity to specifically limit pathogenic CAFs could bypass the limiting drug responses observed in broad-spectrum CAF-targeting therapies, and has great potential to synergise with other therapies to deliver robust therapeutic responses.

## Author Contributions

All authors listed have made a substantial, direct, and intellectual contribution to the work, and approved it for publication.

## Funding

M. Ernst is a recipient of Investigator and Program Grant support from the National Health and Medical Research Council (NHMRC) Australia (1173814).

## Conflict of Interest

The authors declare that the research was conducted in the absence of any commercial or financial relationships that could be construed as a potential conflict of interest.

## Publisher’s Note

All claims expressed in this article are solely those of the authors and do not necessarily represent those of their affiliated organizations, or those of the publisher, the editors and the reviewers. Any product that may be evaluated in this article, or claim that may be made by its manufacturer, is not guaranteed or endorsed by the publisher.
